# The Complete Plastid Genomes of the Two ‘Dinotoms’ *Durinskia baltica* and *Kryptoperidinium foliaceum*

**DOI:** 10.1371/journal.pone.0010711

**Published:** 2010-05-19

**Authors:** Behzad Imanian, Jean-François Pombert, Patrick J. Keeling

**Affiliations:** Department of Botany, University of British Columbia, Vancouver, British Columbia, Canada; Innsbruck Medical University, Austria

## Abstract

**Background:**

In one small group of dinoflagellates, photosynthesis is carried out by a tertiary endosymbiont derived from a diatom, giving rise to a complex cell that we collectively refer to as a ‘dinotom’. The endosymbiont is separated from its host by a single membrane and retains plastids, mitochondria, a large nucleus, and many other eukaryotic organelles and structures, a level of complexity suggesting an early stage of integration. Although the evolution of these endosymbionts has attracted considerable interest, the plastid genome has not been examined in detail, and indeed no tertiary plastid genome has yet been sequenced.

**Methodology/Principal Findings:**

Here we describe the complete plastid genomes of two closely related dinotoms, *Durinskia baltica* and *Kryptoperidinium foliaceum*. The *D. baltica* (116470 bp) and *K. foliaceum* (140426 bp) plastid genomes map as circular molecules featuring two large inverted repeats that separate distinct single copy regions. The organization and gene content of the *D. baltica* plastid closely resemble those of the pennate diatom *Phaeodactylum tricornutum*. The *K. foliaceum* plastid genome is much larger, has undergone more reorganization, and encodes a putative tyrosine recombinase (*tyrC)* also found in the plastid genome of the heterokont *Heterosigma akashiwo*, and two putative serine recombinases (*serC1* and *serC2*) homologous to recombinases encoded by plasmids pCf1 and pCf2 in another pennate diatom, *Cylindrotheca fusiformis*. The *K. foliaceum* plastid genome also contains an additional copy of *serC1*, two degenerate copies of another plasmid-encoded ORF, and two non-coding regions whose sequences closely resemble portions of the pCf1 and pCf2 plasmids.

**Conclusions/Significance:**

These results suggest that while the plastid genomes of two dinotoms share very similar gene content and genome organization with that of the free-living pennate diatom *P. tricornutum*, the *K. folicaeum* plastid genome has absorbed two exogenous plasmids. Whether this took place before or after the tertiary endosymbiosis is not clear.

## Introduction

The path of plastid evolution has been neither simple nor linear, but rather full of twists and turns. After the divergence of glaucophytes, red and green algae following primary endosymbiosis, plastids spread by the secondary and tertiary uptake of these eukaryotic algae by new eukaryotic hosts [1], [2], [3], [4]. Each of these endosymbiotic events involved a massive loss of genes from the symbiont as well as a large scale transfer of other genes to its new host. In the primary endosymbiosis this meant gene transfers from the ancient cyanobacterium, whereas in secondary and tertiary endosymbioses most gene transfer would be from the nucleus of the endosymbiont alga to the nucleus of its new host [5], [6], [7]. The products of many of these genes would be targeted to the plastid, which necessitated the development of a new protein targeting system to direct the protein products back to their correct location [8], [9].

These processes have been most thoroughly studied in primary and secondary plastids, but tertiary endosymbioses add another layer of complexity to the process. In tertiary endosymbiosis an alga with a secondary plastid is taken up by another eukaryote, and to date the only lineage known to take up tertiary plastids is dinoflagellates, where tertiary plastids derived from three different lineages are known: *Karenia* and *Karlodinium* species with plastids derived from a haptophyte [7], [10]; *Dinophysis* species with cryptophyte derived-plastids [11], [12], [13]; and a small but growing group of dinoflagellates harboring a diatom endosymbiont [14], [15], [16], [17], [18], which we refer to as dinotoms. By dinotoms, we will refer to the whole biological system that includes both the dinoflagellate host and the diatom endosymbiont.

Dinotoms are widely distributed in both freshwater and marine environments and some, most notably *Kryptoperidinium foliaceum* and *Peridinium quinquecorne*, form blooms with occasional harmful effects [19], [20], [21]. The Dinoflagellate host component are currently divided into at least five distinct genera, *Kryptoperidinium*, *Durinskia*, *Peridinium*, *Gymnodinium*, and *Galeidiniium* [14], [15], [16], [17], [18], [22], while the endosymbiont components have been shown to originate from three different diatom lineages, one pennate [23], [24], [25], [26] and two centric [27], [28].

In haptophyte and cryptophyte endosymbiont-containing dinoflagellates, the endosymbiont has reduced to the point that only the plastid itself remains. In contrast, the diatom endosymbionts in dinotoms have preserved more of their genetic and cellular identity than any other secondary or tertiary plastid. The endosymbiont has lost some characters such as its cell wall, motility, and the ability to condense its chromosomes normally or divide mitotically [14], [29], [30], but it retains a large nucleus and the nuclear genome, mitochondria and the mitochondrial genome [24], [31], as well as cytosolic ribosomes, endoplasmic reticulum (ER), and dictyosomes in an extensive cytoplasm that is separated from the host by a single membrane [14], [32]. Despite such unusual degree of character retention, the endosymbiont is permanently integrated within its host, and it is present at all different stages of the life cycle including cell division, sexual reproduction, and cyst formation [30], [33], [34].

The number of plastids in dinotoms varies from one or two (in gametes) to as many as 30 to 40 (in zygotes). Chlorophyll *a*, *c1*, and *c2* are among the plastid pigments found in the best-studied dinotoms, *K. foliaceum* and *Durinskia baltica* [35], [36]. The main carotenoid in the plastids of these two dinotoms is identified as fucoxanthin [35], [36], [37], [38] as expected of a diatom and opposed to peridinin, which is the typical plastid carotenoid in dinoflagellate plastids [39]. The peripherally distributed plastids are enclosed in the endosymbiont ER (which is continuous with the nuclear envelope), and retain thylakoids in stacks of three, girdle lamellae, and an internal pyrenoid [14], [16], [17], [18], [40].

Although tertiary endosymbiosis has been subject to a good deal of investigation in recent years, the actual genomes of tertiary plastids have received little attention, and to date no tertiary plastid genome has been sequenced from any lineage. Here, we describe the complete plastid genomes from two dinotom endosymbionts, *K. foliaceum* and *D. baltica*, in order to investigate the impact of tertiary endosymbiosis on the content and organization of these genomes. By comparing these genomes with each other and with available plastid genomes from free-living diatoms we find that the tertiary endosymbiosis has led to little change in either form or content of the plastid genome. However, the plastid genome of the endosymbiont of *K. foliaceum* is much larger than that of either free-living pennate diatoms or *D. baltica*, apparently due to the acquisition, incorporation, and maintenance of integrase/recombinase-encoding plasmid-like elements that are sporadically distributed in other heterokonts.

## Results

### Genome structure, gene repertoire, and GC content of the *D. baltica* and *K. foliaceum* genomes

The *D*. *baltica* CSIRO CS-38 plastid genome (GenBank: GU591327) assembly contained 18704 Titanium pyrosequencing 454 reads (363 bp average), amounting to 6.8 Mbp, or 58–fold coverage of the genome. The *K*. *foliaceum* CCMP 1326 plastid genome (GenBank: GU591328) assembly included 7274 reads (383 bp average) amounting to 2.8 Mbp, or 20-fold coverage. Over 20 kb of the *D*. *baltica* and 75 kb of *K*. *foliaceum*'s plastid genome sequences were also ascertained by PCR and Sanger sequencing (see Methods).

**Figure 1:**
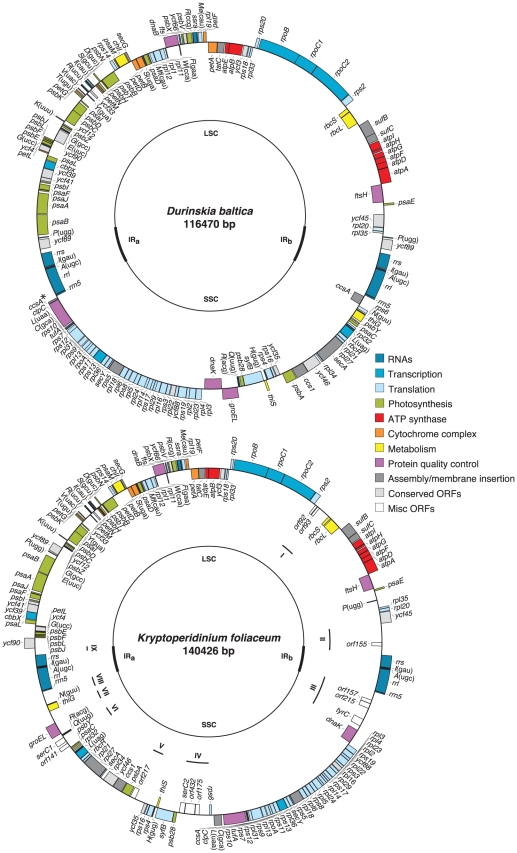
The plastid genome maps of *Durinskia baltica* and *Kryptoperidinium foliaceum*. Functionally related genes are indicated by color and transcriptional direction is indicated by boxes outside the ring (clockwise) or inside the ring (counterclockwise). Genes for tRNAs are indicated by their single letter code. The large single copy (LSC), small single copy (SSC), and inverted repeats (IRa and IRb) are shown on the inner circle. Roman numerals (I-IX) mark the locations of 9 distinct regions in the plastid genome of *K. foliaceum*.

The *D. baltica* and *K. foliaceum* plastid genomes (Figure 1) map as circular molecules divided into large single-copy (LSC) and small single-copy (SSC) regions by the two inverted repeats (IRs), a quadripartite structure that is common to many other algal plastid genomes including the pennate and centric diatoms *P. tricornutum* and *Thalassiosira pseudonana*, respectively [41]. The general characteristics of all diatom and diatom-derived plastid genomes are juxtaposed in Table 1.

**Table 1:** General characteristics of plastid genomes in dinotoms compared to diatoms.

	*Durinskia baltica*	*Kryptoperidinium foliaceum*	*Phaeodactylum tricornutum* a	*Thalassiosira pseudonana* a
Size (bp)				
Total	116470	140426	117369	128814
IR	7067	6017	6912	18337
SSC	39813	56521	39871	26889
LSC	62523	71871	63674	65250
GC content (%)				
Total	32.55	32.4	32.56	30.66
rRNA genes	46.9	47.0	47.2	47.0
tRNA genes	53.5	53.7	53.0	52.6
Other RNAs	27.3	28.3	26.0	25.6
Protein-coding genes	32.4	33.0	32.9	31.5
Intergenic spacer b	22.1	26.5	18.8	16.3
Coding sequence (%) c	86.7	71.9	87.5	85.2
Gene content d				
Total	159	160	162	159
Protein-coding genes	127	128	130	127
rRNA genes	3	3	3	3
tRNA genes	27	27	27	27
Other RNAs	2	2	2	2
Introns	0	0	0	0
Overlapping genes	4	4	4	4
Average intergenic spacer (bp)	94.3	246.7	88.4	108.2
Start codons				
ATG	123	123	124	121
GTG	4	5	5	5
Other	0	0	1 ATT	1 ATA

a Data taken from Oudot-Le Secq *et al* [41].

b Duplicated genes were taken into account (size/number of genes).

c Conserved genes (unique and duplicated) and ORFs were considered as coding sequences.

d Duplicated genes and unique ORFs were not taken into account.

Like other related plastids, both dinotom plastid genomes use standard plastid/bacterial genetic code, with GTG as alternative start codon to ATG. This alternative start codon is found in the same four plastid genes (*rbcS*, *rpl23*, *rps8*, and *rpl3*) in all four diatom and diatom-derived plastid genomes.

The IRs in *D. baltica* are very similar to those of the free-living diatom *P. tricornutum* and feature almost the same gene composition (*trnP*, *ycf89*, *rrs*, *trnI*, *trnA*, *rrl*, and *rrn5*) and size. The slight difference in the size and composition of the IRs in these two plastids is due to the presence of *psbY* in the IRs of *P. tricornutum* instead of partial *ccsA* in the IRs of *D. baltica*. The plastid genome size and gene content of *D. baltica* are remarkably similar to those of *P. tricornutum*. The *D. baltica* plastid genome is only about 900 bp shorter than that of *P. tricornutum*, and the two genomes share 159 genes in common. The *D. baltica* plastid genome encodes 127 protein-coding genes, three rRNAs, 27 tRNAs, a sufficient set for their plastid protein synthesis machinery, one transfer-messenger RNA (tmRNA), *ssra*, and one plastid signal recognition particle RNA, *ffs*. Interestingly, like *P. tricornutum* it has retained *syfB*, encoding a trnF synthetase, which is missing from the plastid genome of *T. pseudonana* but is present in red algal plastid genomes [41]. Only three genes present in the plastid genome of *P. tricornutum* are absent from the *D. baltica* genome: *tsf* (not found in other diatom plastid genomes), *acpP*, and *ycf42*.

In contrast, the *K. foliaceum* plastid genome is considerably larger than the plastid genomes of *D. baltica* and *P. tricornutum*, by about 24 and 23 kb, respectively. The IRs in *K. foliaceum* are shorter than those of *D. baltica* and *P. tricornutum* by almost 1 kb because of the absence of *trnP* and *ycf89* in the *K. foliaceum* IRs, so its larger size is not due to the increased size of the IRs as seen in *T. pseudonana* [41]. Instead, both SSC and LSC in *K. foliaceum* are sizably larger than those observed in other diatoms, owing to the presence of more apparently non-coding DNA (see below) and protein-coding genes. In addition to the same 159 genes found in both *D. baltica* and *P. tricornutum*, the plastid genome of *K. foliaceum* encodes a putative tyrosine recombinase gene, *tyrC*, two putative serine recombinase genes, *serC1* and *serC2*, two smaller ORFs, ORF93 and ORF92 both related to *serC1*, and seven putative open reading frames (ORFs) larger than 150 amino acids (aa), or 15 ORFs if the threshold for annotation is lowered to 100 aa.

### Compactness of dinotom plastid genomes

Like other chromist plastid genomes, the plastid genomes of the two dinotoms possess some of the features of a compact genome. They lack introns, and the same four overlapping pairs of genes found in diatoms [41] are also found in both dinotoms with the identical length of overlap: *psbD*-*psbC*, *atpD*-*atpF*, *sufC*-*sufB*, and *rpl4*-*rpl23* with 53, 4, 1, and 8 nucleotides (nt) overlap, respectively. In addition, *dnaB* and *trnF* have no intergenic spacer in *D. baltica* and *P. tricornutum*, whereas this gene pair is separated by 1 nt in *K. foliaceum*. Similarly, *rpl14* and *rpl24* are separated by a single nt in *D. baltica*, *K. foliaceum*, and *P. tricornutum*.

The plastid genomes of *D. baltica*, *K. foliaceum*, and *P. tricornutum* demonstrate no considerable change in the length of their genes (Figure S1). Out of the common 159 genes, 108 are invariant in length and the sum of all differences between *P. tricornutum* genes and those of *D. baltica* and *K. foliaceum* amount to a mere 199 and 142 bp, respectively (and only 57 bp between *K. foliaceum* and *D. baltica*; Figure S1).

Average intergenic spaces in *D. baltica* (94.3 bp) are only slightly longer than those of *P. tricornutum* (88.4 bp), but in *K. foliaceum* the spacing is more than twice as long (246.7 bp on average) (Table 1). Even when putative ORFs in *K. folicaeum* are brought into account, the average spacing is 180 bp, but more importantly when the average is calculated based only on the 159 shared genes, the average is only 94.1 bp, about equivalent to *D. baltica* and *P. tricornutum*.

### Conserved ordered gene blocks

**Figure 2:**
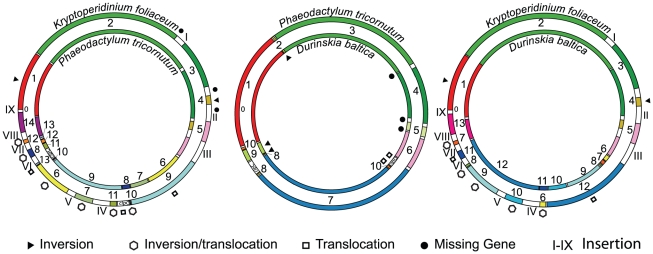
Conserved ordered gene blocks among three plastid genomes. All possible two-way comparisons between plastid genomes of *K. foliaceum, D. baltica*, and *P. tricornutum*. Conserved blocks of genes are indicated by color, inversions are marked by a black triangle, inversions/translocations by a hexagon, translocations by a rectangle, missing genes by a black circle and insertions by Roman numerals I-IX.

To investigate the conservation of genome structure, MAUVE [42] was used to detect gene clusters. Overall, 23 conserved clusters were found in *T. pseudonana*, *P. tricornutum*, *D. baltica*, and *K. foliaceum*. If *T. pseudonana* (a more distantly related centric diatom) is removed from analysis, 14 larger blocks are found. In pairwise comparisons, nine large conserved blocks are shared between *P. tricornutum* and *D. baltica*, 13 between *P. tricornutum* and *K. foliaceum*, and nine between *D. baltica* and *K. foliaceum*. However, taking into account the presence or absence of a single gene between large blocks extends these blocks (to 16 conserved blocks among the three species amounting to more than 108 kb, 10 blocks between *P. tricornutum* and *D. baltica*, 14 blocks between *P. tricornutum* and *K. foliaceum*, and 13 blocks between *D. baltica* and *K. foliaceum*) (Figure 2). The largest block conserved among the three species spans more than 31 kb and includes 46 genes appearing in the same order, encoded on the same strands (*ycf33*, *trnI*, *trnS* … *rpoC1*, *rpoC2*, *rps2*). The largest conserved gene block between *P. tricornutum* and *D. baltica* is about 33 kb and contains 51 genes (*rpl32*, *trnL*, *rbcR* … *rps7*, *tufA*, *rps10*). This conserved gene block is broken into four smaller, dispersed blocks of genes in *K. foliaceum* (*rpl32*-*psbA* and *ycf35*-*psb28*, which are also inverted; *trnQ*-*groEL*; and *dnaK*-*rps10*). There are two small blocks of tRNAs (*trnR*, *trnV*, *trnY*, and *trnL*, *trnC*) that are conserved in three species, but they are inverted in *D. baltica* and *K. foliaceum* with respect to *P. tricornutum*. Similarly, two small conserved blocks of genes (*rpl20*, *rpl35*, *ycf45* and *psbC*, *psbY*) appear in inverted orientation in *D. baltica* with respect to the other two species.

To see how the organization of blocks of shared genes might have evolved, GRIMM [43] was used to identify 14 inversions in the transition of the three plastid genomes of *P. tricornutum*, *D. baltica*, and *K. foliaceum*. If *T. pseudonana* is added, 23 inversions are required. In pairwise analyses, GRIMM also proposes 6 inversions for *P. tricornutum* and *D. baltica*, 9 for *P. tricornutum*, and *K. foliaceum*, and 8 for *D. baltica* and *K. foliaceum*.

Closer manual inspections reveal that compared to the plastid genome of *P. tricornutum* fewer rearrangements of the conserved gene blocks distinguish *D. baltica* from *K. foliaceum*: only three inversions (blocks 2, 8, and 9) and two translocations (block 10 and *clpC* gene) are detected in *D. baltica* versus two inversions (blocks 1, 4), six inversions/translocations (blocks 10, 11, 7, 6, 8, and 12) and three translocations (blocks 9, 13, and *clpC* gene) in *K. foliaceum* (Figure 2). Compared to the plastid genome of *D. baltica*, *K. foliaceum* shows two inversions (blocks 1, 4), five inversions/translocations (blocks 6, 10, 9, 8, and 7) and two translocations (blocks 12, and 11).

All the three missing genes from the plastid genomes of *D. baltica* and *K. foliaceum*, present in *P. tricornutum*, are located in its LSC region. Curiously, however, most of the rearrangements seem to have occurred in the SSC regions of the plastid genomes of *D. baltica* and *K. foliaceum* (Figure 2).

### Low gene density regions of the *Kryptoperidinium foliaceum* plastid genome

There are nine distinct regions (labeled with Roman numerals in Figures 1 and 2) within the *K. foliaceum* plastid genome that have a low gene density and do not show any similarity to *D. baltica*, *P. tricornutum*, or *T. pseudonana*. Six of the nine regions are dispersed within the SSC (regions III-VIII, totaling to more than 17 kb) and three within the LSC (regions I, II, and IX, amounting to about 7.5 kb). All four junctions of the IRs with the SSC and LSC include such regions: II and IX at the boundary of IRa and LSC, and III and VIII at the junction of IRb and SSC. These nine distinct regions collectively amount to more than 24 kb ranging in size from 905 bp (region IX at the boundary of IRa and LSC) to 4852 bp (region III at the junction of IRb and SSC) with an overall GC content of 30.4%, which is 2% lower than the GC content of the genome as a whole (Table 1), and 2.4% lower than the rest of the genome.

Interestingly, regions I and III are each bounded by two imperfect palindromes. A 35 bp palindrome is located near the *rps2* gene and a 44 bp palindrome is located at its other end, near *rbcS*. Region III is similarly bounded by two palindromic sequences: a 25 bp sequence near the *rrn5* gene and a 42 bp sequence near *dnaK*. Another 32 bp palindrome is close to one end of region V (near *psbA*).

### *tyrC* in *K. foliaceum* and *Heterosigma* *akashiwo*

The *tyrC* gene located in region III shows strong similarity to a putative site-specific tyrosine recombinase protein (TyrC) encoded within the plastid genome of the raphidophyte heterokont *H. akashiwo* [44]. The conceptual translation of *tyrC* also shows similarity, albeit much weaker, to putative integrase/recombinase proteins encoded in the plastid genome of the chlorophycean alga *Oedogonium cardiacum* and in the mitochondrion of the charophyte *Chaetosphaeridium globosum*. As revealed by NCBI Conserved Domain Database (CDD) searches [45], the *K. foliaceum* TyrC has conserved all the major catalytic, active, and DNA-binding sites required by this protein for integrase/recombinase activity, including His 250 and the four invariably conserved sites Arg 145, Arg 253, Lys 172, and Tyr 285 (Figure S2) [46], [47], [48]. RT-PCR was performed on *tyrC* and the amplicon sequenced (data not shown), confirming that this gene is transcribed and most likely expressed in the *K. foliaceum* plastid genome.

### Similarity between the *K. foliaceum* plastid genome and pCf1 and pCf2 plasmids in *Cylindrotheca fusiformis*

A total of five ORFs (*orf141*, *serC1* (*orf205*), *serC2* (*orf212*), *orf93*, and *orf92*) in the *K. foliaceum* plastid genome show strong similarity to ORFs found in the pCf1 and pCf2 plasmids of the pennate diatom *C. fusiformis*. Each of these two plasmids includes several ORFs, two pairs of which share considerable similarity (ORF217 of pCf2 and ORF218 of pCf1 with almost 80% aa identity and ORF484 of pCf2 and ORF482 of pCf1 with 54%) [49]. *K. foliaceum* ORF141 (region VI) shares 57% and 47% aa identity with ORF484 (aa 186 to aa 324) from pCf2 and ORF482 from pCf1 plasmid, respectively. The *K. foliaceum* SerC1 shares 76% and 66% aa identity with ORF218 from pCf1 and ORF217 from pCf2, respectively, while SerC2 displays 60% aa identity with *C. fusiformis* ORF218 and 61% with ORF217. Interestingly, SerC1 and SerC2 share less similarity to each other (57% of aa identity) than they do with *C. fusiformis* ORF218 and ORF217, and *serC2* also shares a single codon insertion specifically with ORF217. *K. foliaceum orf93* and *orf92* (region I) appear to be truncated versions of the *C. fusiformis* ORF218, corresponding to amino acids 1 to 93 and 117 to 206, respectively. The two *K. foliaceum* fragments are separated from each other by 69 bp, the conceptional translation of which shares 87% identity with *C. fusiformis* ORF218 amino acids 95 to 116, however this region contains two stop codons suggesting it is a pseudogene.

CDD searches [45] reveal that SerC1 and SerC2 in *K. foliaceum* have retained almost all the catalytic, DNA-binding, presynaptic, and synaptic residues found in other site-specific serine recombinases (Figure S3). Once again, RT-PCR (data not shown) showed that both *serC1* and *serC2* are transcribed and most likely expressed in *K. folicaeum*.

In addition to the five abovementioned ORFs, a number of dispersed non-coding stretches of DNA in several distinct regions of the *K. foliaceum* plastid genome show strong similarity to the *C. fusiformis* pCf1/pCf2 plasmid sequences. A 350-bp sequence in region III of *K. foliaceum*'s plastid genome (Figure 1) shows 92% nt identity to a portion of ORF484 (corresponding to aa 322 to 431) from pCf2. This 350-bp sequence does not include any ORF but contains two stop codons in the same frame that shows similarity to ORF484. Similarly, in region VI about 600 bp immediately downstream of ORF141 shows 71% nt identity to *C. fusiformis* ORF484 and region IV contains 240 bp, 110 bp, and 120 bp sequences with strong similarity to non-coding regions of pCf2 (72%, 72% and 74% nt identity). There is also a 156 bp sequence in region VIII with strong similarity (66% nt identity) to the non-coding region at the end of pCf1.

We also searched the non-plastid 454 sequence data (both assembled and singleton reads) for potential plasmids similar to those of *C. fusiformis*, however, none were found. Since ORF218 and ORF482 are close to each other in pCf1 and similarly ORF217 and ORF484 are close in pCf2, we also designed outward primers for the two corresponding *K. foliaceum* ORFs (ORF205 or *serC1* and ORF141). All attempts to PCR amplify a small product were unsuccessful.

## Discussion

### The divergent evolution of two tertiary plastid genomes of diatom origin

The plastid genomes of the tertiary endosymbionts of *K. foliaceum* and *D. baltica* share numerous common features with those of free-living diatoms, including gene content, ordered gene blocks, and overall genome structure. Especially striking is the similarity to the pennate diatom *P. tricornutum*, with which they share more than 108 kb of syntenic gene clusters, reconfirming the pennate diatom ancestry for these endosymbionts also suggested by molecular phylogeny [23], [24], [25], [26], [27]. Recent phylogenetic analyses suggest a particularly close relationship with the genus *Nitzschia* [24], [26], [27]. Unfortunately, at present the only pennate diatom plastid genome known is from the more distantly related *P. tricornutum*. Considering the high degree of conservation between its plastid genome content, composition, and organization and those of *K. foliaceum* and *D. baltica*, we suggest the plastid genome of closer free-living pennate diatom relatives will reveal even fewer structural changes have taken place since the tertiary endosymbiosis.

Notwithstanding the high degree of conservation between the tertiary plastid genomes and their free-living relative *P. tricornutum*, the plastid genome of *K. foliaceum* is different in one interesting respect. Its genome is more than 23 kb larger than those of its close relatives and the majority of the additional sequence falls into a handful of specific regions. Most of this sequence shows no strong similarity to known sequences, but a few regions share a strong similarity to the plasmids pCf1 and pCf2 in the pennate diatom *C. fusiformis*. The genome also encodes two site-specific serine recombinase genes also shared with those plasmids, as well as a site-specific tyrosine recombinase gene present in the plastid genome of another heterokont, the raphidophyte *H. akashiwo*.

Earlier hybridization experiments suggest that either pCf1/pCf2 or plasmids with considerable sequence similarity existed in three strains of *C. fusiformis*, in one of three strains of *C. closterium*, in *Nitzschia angularis*, and in *N. curvilineata* [49], [50], but no sequence data were available to indicate the possible sites of hybridization or integration of such plasmids with the plastids. In *K. foliaceum* we find no evidence for the presence of intact plasmids, only the putative *serC1* and *serC2* genes and some degenerated fragments integrated into the plastid genome. Overall we can conclude that both plasmids were present in an ancestor of *K. foliaceum*, and fragments of both have persisted by integration into the plastid genome in *K. foliaceum*, but not *D. baltica*.

While the two serine recombinase genes in the *K. foliaceum* plastid genome clearly originated from plasmids and probably functioned in spreading those plasmids, the origin of the tyrosine recombinase/integrase is less clear. TyrC in *H. akashiwo* has been speculated to be involved in converting multimeric plastid molecules to monomeric forms [44], similar to what other recombinases do in certain bacteria with circular chromosomes. In *Escherichia coli*, homologous recombination of the two sister chromatids results in formation of a chromosome dimer, and reversion of the dimer to monomers before cell division is accomplished through the functions of two related recombinases, XerC and XerD [51], [52], [53]. These two proteins break and re-ligate DNA strands at conserved specific binding sites (dif), found in the chromosomal segregation region. The dif sites are usually 28 bp long with two arms, 11 bp each, separated by 6 bp in the center [51], [52], [53]. We did not find any sequences similar to the proposed dif sites of *H. akashiwo* or other known bacteria [44], but whether TyrC in *H. akashiwo* and *K. foliaceum* bind dif sites similar to those of bacterial or viral recombinases, and whether this protein is active in conversion of multimeric forms of plastid genome or even those of the plasmids to monomers are unknown. However, the conservation of all the active sites in the conceptual translation of *K. foliaceum tyrC* (Figure S2) and its transcription both imply that the protein is functional. The presence of palindromic sequences at the boundaries of at least two of the distinct regions (I and III, the latter of which also contains a recombinase gene) in the plastid genome of *K. foliaceum* further suggests these elements may remain mobile by some means that generates such ends during movement/replication.

### The ancestral state of the tertiary endosymbiont genome

Despite the more recent common ancestry of the *D. baltica* and *K. foliaceum* plastids, the *D. baltica* and *P. tricornutum* plastid genomes share a much greater overall similarity in structure, in large part due to the presence of plasmid-associated sequences in *K. foliaceum*. Which of these two tertiary endosymbionts better represents the state of their common ancestor is not entirely clear. On one hand, the close similarity between the genomes of *D. baltica* and *P. tricornutum* might suggest this represents the ancestral state and that the genome of *K. foliaceum* subsequently acquired plasmids (which are selfish and frequently mobile elements) leading to its expansion and reorganization. On the other hand the plasmids are known to exist in some form in other pennate diatoms that are more closely related to the tertiary plastids, most notably some *Nitzschia* species [54]. If they were in the ancestor of the tertiary endosymbionts then *D. baltica* would have to have ridded itself of all evidence of both plasmids to revert to a highly similar form as *P. tricornutum*. Both explanations, the multiple movements of plasmids between close relatives or the complete loss of plasmids in certain lineages, are consistent with the seemingly sporadic interspecies and intraspecies distribution of these plasmids in diatoms: only 5 out of 18 examined diatom species and only 1 out of 3 strains of the pennate diatom *C. closterium* are suggested to have similar plasmids [54]. Perhaps the most likely explanation is that the ancestor possessed unintegrated plasmids and a plastid genome with a structure highly similar to that of *P. tricornutum* and *D. baltica*. In *K. foliaceum* the plasmids would have integrated into the main plastid genome, degenerated, and promoted the reorganization of many gene blocks, whereas in *D. baltica* all traces of the plasmids were lost, which is not unlikely if they never integrated into the plastid genome.

### Conclusions

Here we describe the first completely sequenced plastid genomes from tertiary endosymbionts, specifically the diatom-derived plastids of two dinoflagellates, *D. baltica* and *K. foliaceum*. Both genomes have retained many characteristics of the ancestral, free-living diatom, including elements of genome structure, gene content, and ordered gene clusters. The plastid genome of *K. foliaceum* is much larger than that of *D. baltica*, and contains a site-specific tyrosine recombinase gene also found in the heterokont *H. akashiwo*, and the incorporation, maintenance, and degradation of genetic material from two similar plasmids found in other pennate diatoms, which have resulted in the addition of two site-specific serine recombinases.

## Materials and Methods

### Strains and culture conditions

Cultures of *Durinskia baltica* (*Peridinium balticum*) CSIRO CS-38 and *Kryptoperidinium foliaceum* CCMP 1326 were obtained respectively from the CSIRO Microalgae Supply Service (CSIRO Marine and Atmospheric Research Laboratories, Tasmania, Australia) and from the Provasoli-Guillard National Center for Culture of Marine Phytoplankton (West Boothbay Harbor, ME, USA). *D. baltica* cultures were maintained in GSe medium at 22°C (12:12 light:dark cycle) whereas *K. foliaceum* cultures were maintained in F/2-Si medium under the same conditions.

### DNA and RNA extractions, PCR, RT-PCR, and DNA fractionation and precipitation

Cells were collected and ground as described previously [31]. Ground cells were lysed in 50 mM Tris-Hcl, 100 mM EDTA, 100 mM NaCl, pH 8.0 in the presence of β-mercaptoethanol (2%), SDS (2%) and proteinase K (300 µg/ml) at 50°C for 1 hour. In case of *D. baltica*, 6 phenol and 1 phenol/chloroform extractions were performed, whereas for *K. foliaceum*, 3 phenol, 1 phenol/chloroform, and 2 chloroform extractions were conducted. Organellar A+T-rich DNA was separated from nuclear DNA using CsCl gradient density centrifugation. The initial CsCl reflective index was adjusted to 1.3995 and 1.4000 for *D. baltica* and *K. foliaceum* respectively and Hoechst 33258 (Invitrogen, Carlsbad, CA, USA) was added to the solution (100 µg/ml for *D. baltica* and 120 µg/ml for *K. foliaceum*). Ultracentrifugation was conducted in a Beckman L8 80 M ultracentrifuge, using a VTi 80 (Beckman) rotor at 55000 rpm and 20°C for 22 and 20 hours for *D. baltica* and *K. foliaceum*, respectively. The extracted A+T-rich satellite bands were washed 4 times with CsCl/TE buffer-saturated isopropanol to remove the Hoechst dye. The DNA was precipitated from CsCl as described previously [55] and eluted in Tris HCl pH 8.0. The purified DNA was amplified using the REPLI-g mini kit (Qiagen, Missisauga, ON, Canada). The total genomic DNA from both species used for the PCR reactions was obtained after 2 phenol, 1 Phenol:Chloroform:Isoamyl Alcohol (25:24:1), and 2 chloroform extractions and ethanol precipitation. Total RNA extraction and RT-PCR were carried out as described previously [31] using the following primers: tyrC_F, CCATAACTGCGTAATATAGCCG, tyrC_R, TCTGAAGGAATTAAATCTAATCAAGG, serC1_F, CCAGTTAACTTGCTACTGTCGG, serC1_R TTGGCTCTGCTGCTAACG, serC2_F TGTGTCTTCAAAGTCACAAGAGG, and serC2_R AACTAATCGGTTATATGGTATGTAATTCA. PCR was performed using the EconoTaq PLUS GREEN kit (Lucigen, Middleton, WI, USA).

### Genome sequencing

The *D. baltica* and *K. foliaceum* plastid genomes were sequenced using massively parallel GS-FLX DNA pyrosquencing (Roche 454 Life Sciences, Branford, CT, USA). The GS-FLX shotgun libraries and pyrosequencing using the GS-FLX Titanium reagents were carried out at the Génome Québec Innovation Centre. The Newbler *de novo* assemblies edited and re-assembled with CONSED 19 [56]. Plastid sequences in assembled and unassembled sequence pools were identified by BLAST searches [57]. Ambiguous pyrosequencing homopolymer stretches in the assemblies were verified by PCR/Sanger sequencing, which invariably yielded sequence that preserved the open reading frame. The only exceptions were fragments of plasmid-derived genes in the *K. foliaceum* plastid genomes that are concluded to be pseudogenes.

### Genome annotation and analysis

Genes were identified by DOGMA searches [58] and by BLAST homology searches [57] against the NCBI nonredundant database (http://www.ncbi.nlm.nih/BLAST), and annotated using Artemis 11 [59]. Protein-coding genes were identified using GETORF from EMBOSS 6.0.1 [60] and ORFFINDER at NCBI, with start codons ascertained by comparison with known homologues. Positions of tRNA-encoding genes were determined with tRNAscan-SE [61]. Ribosomal and miscellaneous RNA-encoding genes were annotated by comparison with *P*. *tricornutum* and *T. pseudonana* homologues. Repeated elements were searched for using PipMaker [62], REPuter [63], and FUZZNUC from the EMBOSS package [60]. Physical maps were generated using GenomeVx [64] and further edited manually. Conserved gene clusters between the *D. baltica*, *K. foliaceum* and *P*. *tricornutum* plastid genomes were identified using MAUVE [42] and by visual inspection of the physical maps. Hypothetical gene inversions between the 159 genes that are shared between the three genomes were examined using GRIMM [43]. Translocations were identified by manual inspections and defined as homologous portions of genomes (*i.e.* a gene or a conserved blocks of genes) appearing at different loci in the same orientation.

## Supporting Information

Figure S1 Gene size differences. Pairwise comparisons of gene size differences (larger than 5 bp in at least 2 of the 3 species) in the plastid genomes of *Phaeodactylum tricornutum* (Pt), *Durinskia baltica* (Db), and *Kryptoperidinium foliaceum* (Kf).
(0.92 MB EPS)

Figure S2 Conserved residues found in TyrC. The conserved residues found in the TyrC recombinase encoded in the plastid genomes of *Kryptoperidinium foliaceum* and other site-specific tyrosine recombinases. The sequences of *Heterosigma akashiwo*'s TyrC were manually added to the Conserved Domain Database (CDD) alignment for *K. foliaceum*'s TyrC. The conserved residues with specific functions are marked with a number sign (#) above the alignments. Shaded residues indicate invariable sites among all the recombinases in the alignment, and the long rectangular boxes highlight conserved sites among all the recombinases in the alignment except one.
(2.70 MB EPS)

Figure S3 Conserved residues found in SerC1 and SerC2. The conserved residues found in the SerC1 and SerC2 recombinases encoded in the plastid genomes of *Kryptoperidinium foliaceum* and other site-specific serine recombinases. The sequences of SerC2 were manually added to the Conserved Domain Database (CDD) alignment for SerC1. The conserved residues with specific functions are marked with a number sign (#) above the alignments. Shaded residues indicate invariable sites among all the recombinases in the alignment, and the long rectangular boxes highlight conserved sites among all the recombinases in the alignment except one.
(1.74 MB EPS)
